# *Insects* – An Open Access Journal of Entomology

**DOI:** 10.3390/insects1010001

**Published:** 2010-07-02

**Authors:** Brian T. Forschler

**Affiliations:** *Founding Editor-in-Chief*, Department of Entomology, College of Agricultural and Environmental Sciences, University of Georgia, Athens, GA 30602, USA; E-Mail: bfor@uga.edu

Conventional thought would suggest that there are sufficient venues for the publication of entomological scientific inquiries and begs the question: “Why begin another outlet for insect-related scientific comment?” *Insects* is a response to the pressing global thirst for information and an acknowledgement of the potential of electronic media. Dissemination of information is critical to growing the global knowledge base through easy and open access. This journal is a celebration of the diversity of insects, other arthropods, and their relationship with the human condition, as well as the environment.

Representatives of the *Phylum Arthropoda* are omnipresent on every continent and ocean; over 60% of the planet’s currently estimated 1.9 million animal species are from this *phylum*. This preponderance of presence has influenced human societies since the dawn of time, as evidenced by mention in the folklore, as well as in the writings or products of poets, musicians, artisans, and philosophers from every country on earth. Arthropods have influenced the path of societal histories as vectors of disease, while individual psyches are likewise touched along a continuum of emotions from admiration to fear. Arthropods are an element of the human diet by direct consumption or from the ‘fruits’ of plant fertilization. Economic impacts beyond food production are exemplified by the annual, worldwide output of 30 million kilograms of silk, and one could count the cost of management products and services as another economic influence arthropods have on the everyday quality of the modern human experience.

Biological diversity is intimately linked to arthropod diversity, which is known to influence ecosystem stability, nutrient cycles, trophic structures, plant production, and habitat provisioning. The search for new medicines, industrial products, and agricultural innovations is aided by species diversity and investigating diversity is critical for that quest. Arthropods have been used as models for the study of a wide assortment of questions, ranging from the metaphysical to the molecular. Arthropods have been the subject of research on immune response, herbivory, consensus decision making, predation, mutualism, parasitism, competition, embryology, mimicry, genetics, natural selection, endocrinology, systematics, anatomy, symbiosis, disease transmission, and neurobiology, to name but a few. The practical implications of this research range across disciplines while influencing food production, industrial products, medicinal remedies, soil and water quality, environmental monitoring, energy production, landscape utilization, and building design.

The journal *Insects* is intended to provide an Open Access outlet for research results supporting the global community that works with arthropods as the subject of their investigations. The topics considered for publication will reflect a diversity of topics from a variety of scientific arenas while maintaining the highest rigor of the peer-review process to ensure quality reading for interested stakeholders, including the interested general public. Special issues will be determined and dedicated to specific and timely topics, as suggested by interested authors and editors. The weight of 14 years of publishing experience provided by MDPI [1], based in Basel, Switzerland, are an invaluable aid to realizing the success of this journal. MDPI boasts a professional editorial staff currently publishing more than 30 peer-reviewed journals, of which all major journals are already covered by the Science Citation Index (Web of Science) and Scopus. Long-term archiving is guaranteed through the immediate deposit of published articles with the Swiss National Library (SNL). The dedication of the editorial staff and their assistance with author submissions are critical elements of this journal that will be enjoyed by all contributing authors, and will most certainly aid the acceptance of *Insects* by the scientific community.

The editorial board is committed to ensuring the quality of submissions accepted for publication in *Insects*. Our collective goal is to be recognized as the publication outlet for high quality, cutting edge, research on a broad range of topics in this diverse field. That goal is, however, dependent on authors with a similar dedication to their work that dare challenge accepted ideas and who wish to stimulate discussion and further inquiry by publication in a high profile, globally accessible format. We therefore invite you to send us your original research articles, reviews, and short communications for peer-review and possible publication in *Insects*, as well as to send us your suggestions for potential special issues on hot topics in entomology. Your contributions are essential to realizing the potential for this journal as the source for adding important information to the worldwide scientific knowledge base.
